# Surgical management of low grade isthmic spondylolisthesis; a randomized controlled study of the surgical fixation with and without reduction

**DOI:** 10.1186/1748-7161-6-14

**Published:** 2011-07-28

**Authors:** Ziad M Audat, Fayeq T Darwish, Moh'd M Al Barbarawi, Moatasem M Obaidat, Walid H Haddad, Khaldoon M Bashaireh, Ihsan A Al-Aboosy

**Affiliations:** 1 Department of orthopeadic, Level 8A. King Abdullah University Hospital, Jordan University of Science and Technology, Irbid-Amman Street, P.O.box 3030, Irbid, Jordan; 2 Department of Neuroscience/Division of Neurosurgery, Level 7A. King Abdullah University Hospital, Jordan University of Science and Technology, Irbid-Amman Street, P.O.box 3030, Irbid, Jordan

**Keywords:** spondylolisthesis, surgical fixation, reduction, outcome, neural decompression, ODI

## Abstract

**Background:**

spondylolisthesis is a condition in which a vertebra slips out of the proper position onto the bone below it as a result of pars interarticularis defect. The slipped segment produces abnormal positioning of the vertebrae in relation to each other along the spinal column and causes mechanical back pain and neural breach.

**Materials and methods:**

A randomized and double blinded study consisted of 41 patients aged 36-69 years (18 females and 28 males) treated for symptomatic spondylolisthesis between December,2006 and December, 2009. All patients were randomly distributed into two groups I and II. Twenty patients were in Group I; they underwent reduction of the slipped vertebrae by using Reduction-Screw Technique and posterior lumbar interbody fixation (PLIF). Group II consisted of twenty one patients who underwent only surgical fixation (PLIF) without reduction. All patients in this study had same pre and post operative management.

**Results:**

only one case had broken rod in group I that required revision. Superficial wound infection was experienced in two patients and one patient, from group II, developed wound hematoma. The outcome in both groups was variable on the short term but was almost the same on the long term follow up.

**Conclusion:**

surgical management of symptomatic low grade spondylolisthesis should include neural decompression and surgical fixation. Reduction of slipped vertebral bodies is unnecessary as the ultimate outcome will be likely similar.

## Introduction

Lumbar isthmic spondylolisthesis in adults is a frequent pathology that is encountered by spinal surgeon. It affects 5% of populations in the USA [1]. Clinical presentation is usually variable and ranging from mild to severe symptoms and disability which are related to the neural compression. The symptoms are typically related to the biomechanical spinal instability which leads to disc degeneration and lumber canal stenosis that ends with encroachment of nerve roots and thecal sac at the slide level [2-7].

Medical treatment is usually the first line on management. Surgical approaches are preserved to cases with failure of conservative treatment or those with overt neurological deficits. However, Various surgical techniques have been advocated to deal with symptomatic isthmic spondylolisthesis; the main perception of these surgical techniques focused on spinal fixation and neural decompression [8-11].

Reduction of the slipped vertebrae as a part of surgical approach is still debatable. In the current literature, the studies have paid attention to the surgical reduction of the slippage or in situ spinal fixation technique. These studies are lack of comparison between these variable techniques. In this review, we have addressed this subject and designed a prospective randomized controlled study to compare between surgical fixation with and without reduction of the slipped segment.

## Materials and methods

The prospective, randomized and double blinded study has been approved by the ethical committee for human research (IRB) in Jordan University of Science and Technology. The study group consisted of 41 patients and were treated for symptomatic isthmic spondylolisthesis at king Abdullah university hospital between December, 2006 and December, 2009. The study was designed for a period of 24 months and a follow up of 36 months. The inclusion criteria included symptomatic patients with Meyerding grade I and II isthmic spondylolisthesis that evident on plain radiography; patients with a significant neurological deficits or who failed to respond to conservative treatment, at least, for three months, the medical treatment included strong pain killers, physiotherapy, life style modification and body weight reduction. Symptoms are those of severe and chronic low back pain, sciatica pain, sensory disturbances with or without muscle weakness and neurogenic claudication. Exclusionary criteria included; patients with grade III and VI, traumatic spondylolisthesis, neoplastic spondylolisthesis, patients with acute or chronic infection and congenital malformation.

The patient demographics were reviewed and analyzed in a prospective method. Patients who fulfilled the inclusionary criteria were admitted to the hospital for surgical treatment. Pre-operative assessment was carried out on all patients similarly. This included plain and dynamic lumber spine x-rays, lumber spine MRI and routine lab work. Oswestry Disability Index (ODI) was used for pre and post operative disability assessment in all cases[12].

### Study design

To reduce bias and ensure adequacy of surgical management and outcome; all cases were operated upon by one surgeon. On the other hand, the surgical outcome was assessed by a different surgeon who was not aware to the surgical technique used on the evaluated patient. Patient distribution in the study group was alternatively and randomly selected. All Patients in either group had same surgical approach. All patients in both groups underwent neural decompression and surgical fixation; those who had undergone surgica reduction of the slipped vertebrae were stratified in group I. The surgical reduction of the slipped vertebrae was achieved by applying Reduction-Screw Technique with posterior lumbar interbody fixation (CD-Horizon system with Capston interbody Cage/Medtronic)and artificial bone graft (Tricalcium Phosphate) mixed with patient's bone marrow for better biological fusion. Whereas, patients in group II underwent only surgical fixation and neural decompression. They had no reduction of the spondylolisthesis segment.

All patients had an uneventful post operative course. Postoperative plain X-rays of the lumber spine were done on all patients; the site of surgical fixation appeared satisfactory, and they started ambulation with a Lumbosacral support on the first or second postoperative day. Spinal rehabilitation was organized for all cases. All patients were evaluated in the outpatient clinic on regular basis by an independent surgeon as follows 2 weeks, three, six, twelve, twenty four and thirty six months. Further more, lateral and anterioposterior Lumbosacral spine X-rays were considered on all cases for adequate evaluation of the surgical fixation, progression of spinal fusion, presence of adjacent segment and/or pesudoarthrosis. However, computed tomography scan was also considered in certain conditions. Lenke et al radiological criteria was applied on all cases to evaluate their radiological outcome [13].

Data collection and analysis of outcome were completed by an independent surgeon based on the Oswestry Disability Index (ODI) as following:

#### Poor outcome

patients who experienced same preoperative symptoms or the symptoms had worsened up after surgery and there were a significant restriction of their daily life activities.

#### Fair outcome

pain had improved up to 50% compared with the pre operative status but still requiring strong analgesics; mild improvement in sensory and motor symptoms was evident but the patient still had some difficulty with his daily life activities. Patient's satisfaction was around 50-60%.

#### Good

when the patient had a significant improvement in the back pain and sciatica, occasional analgesics were required and they experienced less numbness and parasthesia with a noticeable improvement in weakness. No constraint in daily activities any more. Patient's satisfaction was 60-80%.

#### Excellent

this group included cases with no more pain or neurological deficits. Normal daily life activities and patient's satisfaction was more than 80%.

## Results

A total of 20 patients in group I (3 males and 17 females) ages 39-64 years (average 51.1 years) were treated with surgical fixation, neural decompression and reduction of spondylolisthesis. There were 21 patients in group II (10 males and 11 females) ages 36-64 years (average 50.14 years) were treated only with neural decompression and fixation *in situ *without reduction of the slipped vertebrae. There was no significant difference between two groups in regards to the clinical presentation, clinical findings and pre operative co morbidities. (Table 1) shows the demographic distribution and characteristics of each group.

**Table 1:** The demographic distribution and clinical presentation of both subgroups.

	Reduction Group I N = 20	%	fusion in situ Group II. N = 21	%
Age	39-64		36-64	

average	51.1		50.14	

Total	21	51.8	20	48.2

male	3		10	

female	17		11	

Severe low back pain				

	21	100	20	100

Radiating or claudicating Pain:				

	20	100	21	100

Unilateral	8	40	7	33.3

Bilateral/cramps	12	60	14	66.7

Sensory disturbance	10	40	8	47.6

Muscle weakness	7	35	6	28.6

History of medical co morbidities (DM. HTN)	9		10	

There were no difference in the operative and post operative co morbidities in both group and that included; pedicle fracture, minor dural tear which was repaired instantly and none of them experienced post operative CSF collection or leak. (6% vs. 5.8% respectively (p-value = 0.520)). However, a few early post-operative complications were encountered such as; superficial wound infection, wound hematoma, post operative transient sciatica pain, as a result of intraoperative nerve root manipulation was seen frequently; pain almost improved with time (frequency in group I and II 22% vs. 25.0% respectively (p-value = 0.645). A broken rod had only occurred in a case in group II.

The hospitalization stay was between seven and nine days (average 7.3 days) for patients of group I. while it was between eight and ten days (average 8.2 days) for patients in group II. The real time of operation was estimated at average of six hours in each group.

On the long term follow up, there was only a patient from group II who developed pesudoarthrosis which required surgical intervention. Adjacent segment disease was not evident on any case in both groups.

### Statistical review

**Figure 1:**
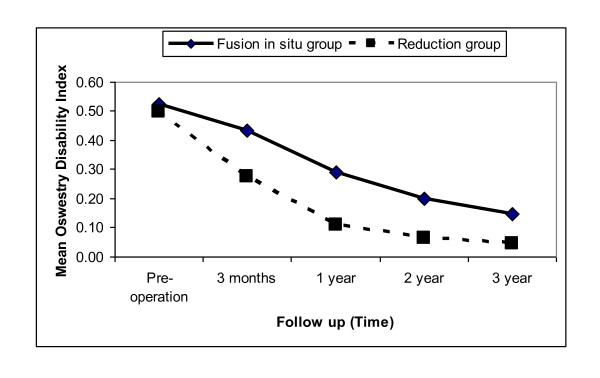
**Mean Oswestry Disability Index**. The scheme shows clearly how the outcome improved on the long term follow up in both subgroups I &II.

Preoperatively, there was no significant difference in mean ODI between patients of group I (mean (SD) = 0.52 (0.10)) and group II (mean (SD) = 0.50 (0.13)) and, p-value = 0.523. Over time, the mean ODI decreased significantly and linearly in both groups with the mean ODI being significantly lower in group I at all period of follow up (P-value <0.005 for each evaluation during follow up) (Figure 1). At the last follow-up evaluation, the mean ODI had decreased from the preoperative value of 0.50 to 0.04 (p-value for trend <0.005) in group I (p-value for trend <0.005) and from 0.52 to 0.15 in group II.

**Figure 2:**
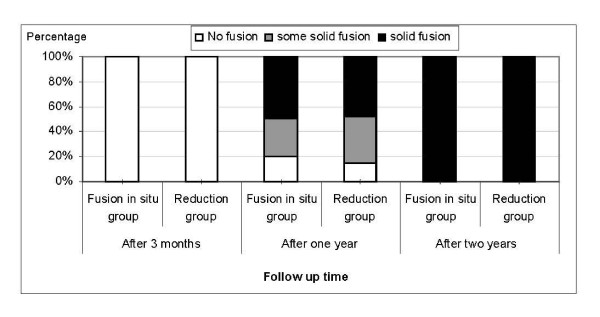
**The diagram reveals the relationship between short and long term follow up and the percentage of improvement in both subgroups**.

Preoperatively, the majority of patients in group I (85%) and group II (81%) had severe disability and the rest had moderate disability (p-value = 0.529). Post operatively 5% from group I and 76.2% patients from group II were estimated to have excellent condition on the day of discharge. While 25% of patients of group I had fair to bad condition on the day of discharge, none of patients from group II had a poor condition (Figure 2). There was 65% of patients had severe disability and 35% had moderate disability on three months' follow up in group I. While none of patients from group II had severe disability; yet 23.8% of cases had remained with mild - moderate disability.

On the long term follow up after two years; all patients of group II had shifted from moderate to mild disability. Saying that, 40% of patients in group I remained with moderate disability.

Further improvement was achieved on the three years' follow up as only 20% of patients continued to have moderate severity in group I. Over the ensuing time, patients in both groups displayed a significant improvement and ended with good to excellent condition (Table 2).

**Table 2:** Demonstrates outcome on the short and long term follow up by using ODI.

**Follow up**	**Reduction Group I**	**Fusion *in situ *Group II**
**On 3 months**		
Bad	0 (0.0)	5 (25)
Fair	2 (9.5)	6 (30)
Good	3 (14.3)	8 (40)
Excellent	16 (76.2)	1(5.0)
**After one year:**		
Bad	0 (0.0)	1(5.0)
Fair	0 (0)	2 (10)
Good	3 (14.3)	11(55)
Excellent	18 (85.7)	6 (30)
**After two years:**		
Bad	0 (0.0)	0 (0.0)
Fair	0 (0)	1(5.0)
Good	2 (9.5)	2 (10.0)
Excellent	19 (90.5)	17 (85.0)
**After three years:**		
Bad	0 (0.0)	0 (0.0)
Fair	0 (0.0)	1 (5.0)
Good	4 (18)	3 (15.0)
Excellent	17(82)	16 (80.0)

**Figure 3:**
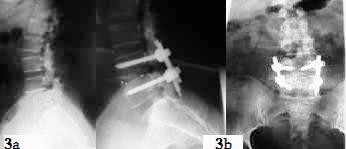
**3a, preoperative lateral plain x-ray which shows L4/5 grade II spondylolisthesis on a young patients who failed medical treatment**. **3b**, post operative anterio-posterior and lateral x-ray on one year follow up. It demonstrates an L4/5 spinal fixation with reduction of the slipped L4/5 segment. Adequate spinal fusion is also noted.

Radiologically, all patients had no fusion evident on the X-rays after 6 months of surgery. However, around 50% of patients, in each group, had a solid fusion after one year (Figure 3a &3b). Most of the patients, 17 from group I and 19 from subgroup II, had solid fusion after two years. Further fusion was achieved on the rest of cases after three years but one case who developed pseudoarthrosis and required revision.

## Discussion

Pedicle screw fixation of spinal column in patients with various spinal disorders has become increasingly popular over the past years particularly for treatment of spondylolisthesis. Management of spondylolisthesis is variable and depends on the underlying pathology. For asymptomatic cases surveillance is the treatment of choice while medical treatment is the first line of management for symptomatic cases. However, surgical treatment is reserved for cases who have failed the medical treatment or to patients with neurological deficits. Various surgical techniques have been used to deal with lumber spine spondylotic spondylolisthesis; basically focused on the concept of spinal fusion. Many have advocated the use of instrumentations with or without neural decompression or only neural decompression without surgical fixation with claim of variable results though [6,7].

The reduction of vertebral step is still a matter of debate. In his study, Mikko et al concluded that patients who had surgical fixation without reduction ended with better outcome compared with patient who underwent surgical reduction and fixation. Yet, This conclusion was drawn on adolescent patients who had severe spondylolisthesis; the other face it might not be applicable on older patients or those with lower grade of spondylolisthesis [14]. The results we concluded in our study have proved that the outcome is almost similar in patients who underwent instrumental fixation along side with neural decompression whether they had reduction of the spondylolisthetic segment or not. This draw a challenge to the results of Mikko et al when his conclusion is being applied on adult patients with low grade spondylolisthesis.

Furthermore, many authors have advocated that correction of sagittal spinal deformity in conjunction with arthrodesis will enhance the spinal biomechanics and results in a nerve root decompression. Besides that, it provides a mechanical protection for the spinal fusion from tensile and shearing forces that may be applied to the adjacent segments and this could prevent an early adjacent segment disease. What makes the slippage reduction in adults amenable and easy is the fact that the slip angle is usually small, and there are no dysplastic changes of adolescent high-grade slips, such as a rounded sacrum or trapezoidal L5 shape. How ever, these facts have been challenged by many authors [15-18].

Adjacent segment disease still a problem that may occur in a high rate on the long term follow up after lumbar spine fixation and estimated at in 36.1% of cases. This may be related to the pre operative abnormal sagittal configuration of the spine rather than to the surgical technique utilized or extension of the spinal fixation or even the existence of degenerative disease. Conversely, it seems that normal sacral inclination is the most important factor for having lower adjacent segment degeneration and Retrolisthesis is the most frequent degenerative type of adjacent segment disease seen [19].

Functional outcome following instrumental spinal surgery for spondylolisthesis in physically energetic patients is crucial. Molinari et al, had reviewed the functional outcome following instrumental surgery and concluded that patients with symptomatic low grade spondylolisthesis could return to high functional life with less back pain following a limited surgical intervention [20].

As a result, There is lack of studies in the literature that compares surgical outcome between patients with low grade spondylolisthesis who underwent surgical fixation with reduction of the vertebral shift and those who underwent only fixation in situ without having the step reduced. Though comparison studies between variable surgical techniques utilized to deal with symptomatic spondylolisthesis have been carried out by many authors. Apparently, the surgical outcome of various techniques used for spinal decompression and instrumental fixation seems to be almost the alike with trivial differences between these techniques in terms of surgical complications, rate of spinal fusion and satisfactory outcome in the short and long term follow up [21,22].

## Conclusion

Surgical treatment of isthmic spondylolisthesis is reserved to the symptomatic case who failed conservative treatment or developed neurological deficits. The gold standard treatment is surgical fixation of the slipped segments with neural decompression. Surgical reduction of spondylolisthesis is a matter of controversy. In this review we concentrated on this uncertainty and concluded that on the short and long term follow up the outcome between patients who underwent surgical fixation and neural decompression with or without correction of the slippage is almost approximated there is no significant difference between these two approaches. How ever, further studies may be needed to confirm our conclusion

## Declaration

The authors declare that they have no competing interests.

## Authors' contributions

ZA: principal surgeon who operated on all cases

FD: participated basically in patients' post operative follow up and clinical assessment main patients

MB: main author

MO: co-author

WH: participated basically in patients' post operative follow up and clinical assessment main patients

KB: participated in data collection and statistical analysis

IA: participated in data collection

All authors have read and approved the manuscript in its final revision.
